# Real-time PCR-based assay to quantify the relative amount of human and mouse tissue present in tumor xenografts

**DOI:** 10.1186/1472-6750-11-124

**Published:** 2011-12-16

**Authors:** Sergio Y Alcoser, David J Kimmel, Suzanne D Borgel, John P Carter, Kelly M Dougherty, Melinda G Hollingshead

**Affiliations:** 1 Biological Testing Branch, Developmental Therapeutics Program, Division of Cancer Treatment and Diagnosis, National Cancer Institute-Frederick, Frederick, MD, USA; 2 Biological Testing Branch, Developmental Therapeutics Program, SAIC-Frederick Inc., NCI-Frederick, Frederick, MD, USA

## Abstract

**Background:**

Xenograft samples used to test anti-cancer drug efficacies and toxicities in vivo contain an unknown mix of mouse and human cells. Evaluation of drug activity can be confounded by samples containing large amounts of contaminating mouse tissue. We have developed a real-time quantitative polymerase chain reaction (qPCR) assay using TaqMan technology to quantify the amount of mouse tissue that is incorporated into human xenograft samples.

**Results:**

The forward and reverse primers bind to the same DNA sequence in the human and the mouse genome. Using a set of specially designed fluorescent probes provides species specificity. The linearity and sensitivity of the assay is evaluated using serial dilutions of single species and heterogeneous DNA mixtures. We examined many xenograft samples at various in vivo passages, finding a wide variety of human:mouse DNA ratios. This variation may be influenced by tumor type, number of serial passages in vivo, and even which part of the tumor was collected and used in the assay.

**Conclusions:**

This novel assay provides an accurate quantitative assessment of human and mouse content in xenograft tumors. This assay can be performed on aberrantly behaving human xenografts, samples used in bioinformatics studies, and periodically for tumor tissue frequently grown by serial passage in vivo.

## Background

Human xenografts implanted and grown in immunodeficient mice are commonly used to expand tumor cell populations for cancer stem cell investigations [1] and to test anti-cancer drug efficacies or toxicities in vivo [2]. It is often assumed any anti-tumor drug activity is due to targeting pathways in the human cells present in the xenograft, and any associated gene expression data is derived from a mostly human cell population. However, as the implanted xenograft grows in the mouse its human stromal cells are replaced by mouse stromal cells [3], influencing its microenvironment and resulting in a tumor xenograft that is a heterogeneous mixture of human and mouse derived cell populations. Each cell type may possess different growth rates and react differently to an administered drug. Further, there are many reliable reports in the literature of human xenografts serially passed in vivo transforming adjacent mouse cells into fibrosarcoma-like malignancies [4-8]. Drugs tested on such samples would generate false and misleading data. How can researchers easily verify that a tumor xenograft sample contains only a relatively small number of contaminating mouse cells?

Over the years, several groups have attempted to answer this question using in situ hybridization [9,10] or immunohistochemical procedures [11]. However, they are slow, labor-intensive endeavors, and are limited by subjective, difficult-to-reproduce measurements. Recently, PCR-based strategies have been developed which take advantage of the ability of species-specific oligomer primers to quickly amplify species-specific genomic DNA sequences. Ono et al [12] were able to differentiate fourteen different species by targeting their relatively abundant and highly conserved mitochondrial DNA sequences. Cooper et al [13] took this a step further by performing fourteen species-specific PCR reactions simultaneously in a single PCR tube ("multiplexing"). Each species-specific amplicon was a unique length, different enough from the others to be successfully resolved on a 4% agarose gel. While these methods are certainly not quantitative, they are fast, easy, and accurate. One way to obtain a more quantitative result is to take advantage of DNA sequencing technology to measure gene length variation by PCR-amplification of several markers across different chromosomes then compare the species-specific differences in relative amplicon lengths [14]. This improved method still requires manually measuring peak heights generated by capillary electrophoresis from an ABI 3100 Genetic Analyzer, a robust but still expensive and uncommon piece of laboratory equipment. This method also relies on the use of multiple genomic loci, many of which are located on or near chromosomal regions known to be deleted or amplified in some human cancers.

Real-Time Quantitative PCR (qPCR) instruments have been used to quantify genomic DNA from multiple species in mixed DNA samples: multiple human targets [15]; rhesus and long-tailed macaques [16]; feline, bovine, equine, and cervid [17]; human, cat, and dog [18]. These machines have become commonplace and a 96-well plate can be processed in 90 minutes or less. Therefore, we set out to develop a multiplex qPCR assay that addresses some of the obstacles that limited previous methodologies. Herein we describe the assay methodology, various quality control analyses, and a survey of human xenograft samples to illustrate the kind of real-world results that can be expected from the assay.

## Results and Discussion

### Initial sequence targeting and validation

**Figure 1:**
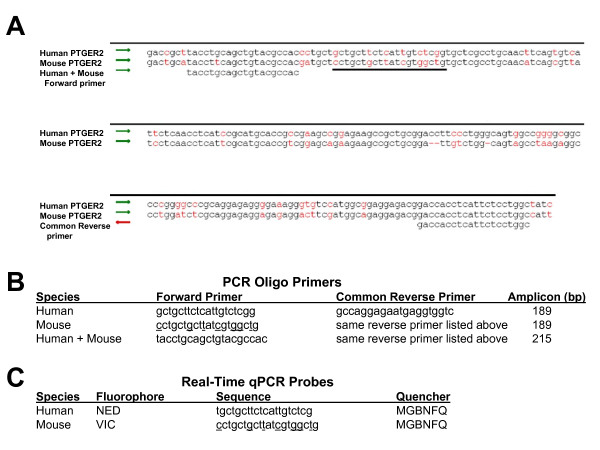
**Relevant species alignment using the PTGER2 gene and the PCR primers and real-time qPCR probe sequences derived from the alignment**. A) The human and mouse PTGER2 DNA sequences are aligned, with red letters signifying non-conserved basepairs. Human+Mouse PCR primers are listed underneath, and a black bar is below the qPCR probe sequence. The reverse-compliment Common Reverse primer sequence is shown to illustrate alignment on the DNA strand shown. B) Qualitative PCR primers are listed, as well as the expected size of the amplified sequence. All sequences listed 5' to 3'. Mouse genome specific base pairs are underlined in the mouse forward primer and mouse probe sequences. C) Real-time qPCR probe sequences are listed next to their fluorophore and quencher.

**Figure 2:**
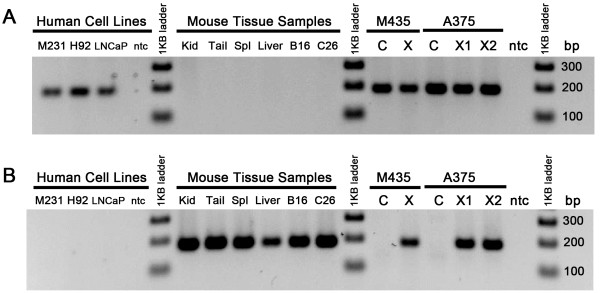
**Species specificity of the qualitative PCR primers**. Panel A was generated using the human-forward and common reverse primers listed in Figure 1B. Only samples containing human cells show the human 189 bp band. Panel B uses the mouse-forward and common reverse primers listed in Figure 1B. Only samples containing genomic mouse DNA show the expected mouse amplicon. As expected, the serially passaged xenograft samples show the presence of both human and mouse DNA, as early as the first in vivo passage (X) and even after ten in vivo serial passages (lanes X1 and X2). Acronyms: M231 (MDA-MB-231 human breast cancer cells), H92 (HOP-92 human lung cancer cells), LNCaP (LNCaP human prostate cancer cells), ntc (no template control), Kid (kidney), Spl (spleen), B16 (B16F10 mouse melanoma tumor), C26 (Colon-26 mouse colon tumor), M435 (MDA-MB-435 human melanoma), A375 (human malignant melanoma), C (cultured cell line), X (xenograft, harvested after one passage in vivo), X1 and X2 (xenograft, harvested after ten serial passages in vivo), bp (base pairs).

We surveyed information from several published reports [19-22] to help identify a chromosomal region that is not known to be frequently duplicated/deleted in human disease or near a recombination hotspot, yet very homologous to the orthologous mouse sequence. The prostaglandin E receptor 2 (*PTGER2*) gene region on human chromosome 14q22 fits these guidelines. We focused on a target region within that gene which has high homology between human and mouse sequences allowing us to design primers which could bind both sequences equally well and amplify the homologous sequences (see Figure 1A). For the species-specific qualitative PCR, we designed species-specific forward primers which contain seven non-homologous base pairs (listed in Figure 1B). Along with the common reverse primer, the species specificity of these primers is illustrated in Figure 2A, where the human-specific forward primer generates a 189 bp band only in samples that are human-only or from xenografts known to contain human material. Similarly in Figure 2B, the mouse-specific forward primer generates the 189 bp band only in samples derived from mouse tissues, mouse-tumor allografts, or human xenografts which invariably contain some mouse tissue. This qualitative, end-point PCR assay can be used to quickly screen xenograft samples to verify the presence of human DNA and confirm that a xenograft has not been replaced by a mouse-only fibrosarcoma.

**Figure 3:**
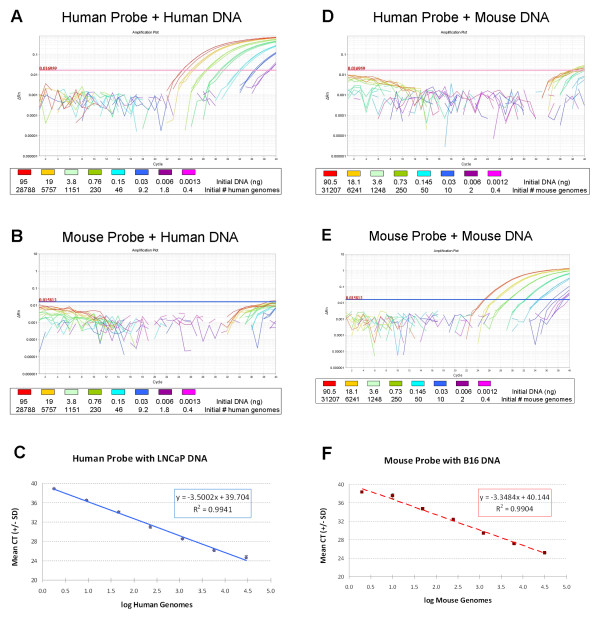
**Representative example of real-time qPCR fluorescent signal traces (ΔRn vs cycles) showing species specificity of the qPCR probes and a species-specific standard curve derived from each data set**. A) The human probe shows robust DNA amplification signals in samples consisting of only LNCaP human prostate carcinoma cell line template DNA. B) In the same LNCaP samples, the mouse probe shows no binding or product amplification. C) Plotting mean CT (+/- SD) vs log human genomes initially present in the qPCR reaction tube, the serial dilution from (A) is linear down to 1.8 human haploid genomes. D) The human-specific probe does not bind to B16F10 mouse melanoma tumor template DNA. E) The mouse-specific probe does show amplification when using B16F10 mouse tumor DNA as a template. F) A standard curve derived from the mean mouse probe CT signal plotted against log mouse genomes present in (E) shows a linear correlation down to 2 initial genomes.

The real-time qPCR assay was designed to estimate the percentage of human and mouse DNA contained in a xenograft or other mixed-DNA sample. The human+mouse forward and common reverse primers were designed to bind the same PTGER sequences on human and mouse genomic DNA (Figure 1A, B). They amplify the same 215 bp DNA sequence, whether the template DNA is from mouse or human sources, minimizing differences in amplification efficiency and primer-binding. Two species-specific Taqman probes, each conjugated with a unique fluorescent tag, were designed to target a non-homologous sequence within the amplified region (Figure 1C). These species-specific probes allow for multiplex reaction chemistry in a single PCR reaction tube, minimizing pipetting error and maximizing the number of samples that can be tested on a single 96-well optical plate. All qPCR data in this manuscript were derived using the probes as described in Figure 1. However, we have also tested the probe sequences with flip-r fluorophores (Human-VIC and Mouse-NED) with no loss of qPCR efficiency or species specificity. To verify the species-specificity of the real-time qPCR assay, we set up reactions containing a known quantity of single species DNA sample along with both qPCR primers and both qPCR probes. Serial dilutions were used to investigate the assay sensitivity and reliability. Figure 3 shows the resulting qPCR traces and the resulting standard curve comparing mean CT to log initial genomes using either the human prostate carcinoma cell line LNCaP or the mouse B16F10 melanoma tumor DNA. As expected, the graphs illustrating the use of human probe + human primers (Figure 3A) or mouse probe + mouse primers (Figure 3E) both show robust sequence amplification from approximately 30,000 down to 2 initial genomes. But the mouse probe does not bind to the amplified human sequence (Figure 3B), and the human probe does not bind to the amplified mouse sequence (Figure 3D), thus confirming the assay's species specificity. Although the variance in CTs increases dramatically when testing the lowest starting DNA amounts (10 genomes or less), the mean CT is still on the linear standard curve down to approximately 2 haploid genomes in both species (Figure 3C and 3F).

### Evaluating mixed DNA samples

**Figure 4:**
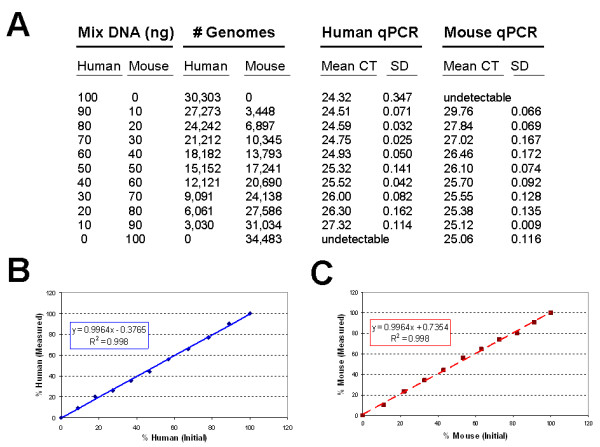
**Example of a serial dilution study to verify linearity in mixed DNA samples**. A) Each sample contains 100 ng total DNA, composed of various ratios of mouse kidney and human MDA-MB-435 melanoma cell line DNA in regular 10 ng increments. The corresponding numbers of initial haploid genomes are calculated, and the mean CT and SD from the qPCR results for reach probe are listed. B) Plotting the measured number of human genomes (y-axis) versus the known number of initial human genomes present in each sample (x-axis) yields a linear curve (blue line) with R**^2 ^**= 0.998. C) Similarly, plotting the measured number of mouse genomes versus the initial number of mouse genomes yields a linear curve with R**^2 ^**= 0.998.

Next, we evaluated the assay using samples containing known amounts of human and mouse DNA at inverse proportions serially diluted such that the total amount of DNA in each sample was 100 ng (Figure 4A). Total genomes of each species were calculated from the known DNA concentrations. Samples were run in triplicate and the mean CTs and standard deviations (SD) are listed in Figure 4A. SDs less than 0.5 are considered acceptable. Genome numbers were calculated as before and converted to "percentage human" and "percentage mouse". These results were compared to the initial number of human genomes in Figure 4B and percent of initial mouse genomes in Figure 4C. There is a linear correlation for both species with R^2 ^= 0.998.

In reality, the ratio of human and mouse DNA in a given xenograft or other mixed DNA sample is initially unknown. We can verify the robustness of the qPCR assay by comparing the initial amount of template DNA in a given sample to the sum of the mass of the measured number of human and mouse genomes. We surveyed 22 human xenograft samples, currently being grown in our facility, comprising different tumor types and various lengths of serial passage in vivo (Table 1). The average percentage of mouse contamination in the tumor xenografts varies widely from 0% (undetectable under current assay conditions) to 87% in MDA-MB-361. Low mouse contamination percentages were found in all in vivo passages, from P1 to P10, and high mouse contamination was found in some P1 passage xenografts, suggesting the length of time spent in vivo is not a significant factor in predicting which xenografts will contain the highest amount of contaminating mouse cells. There was significant variability amongst samples of the same tumor type, as illustrated in the five LOX IMVI tumor samples. Mouse contamination varied in these from 45%-66%, even though all were derived from the same cell culture sample. Important factors that may contribute to the amount of mouse contamination in a xenograft may include the histological type of tumor, how many times it was serially passaged in vivo and how vascularized the xenograft normally becomes. Additional sample collection and analysis is required to investigate this further. The mass of measured human and mouse DNA in each sample can be summed and compared to the initial amount of DNA; the percentage of measured/initial DNA is shown in the far right column in Table 1. This data set was generated by two operators each performing the assay multiple times thus some variance is expected. We routinely find that the percent human and percent mouse measurements are robust from assay to assay, but pipetting variations contribute to the variation in magnitude of the calculated species DNA and adds greatly to the variance sometimes seen in the % of initial DNA calculation. Using only one operator greatly reduces this variance. We have designed this qPCR assay for a 20 μl reaction volume solely as a cost-saving measure. However, using the standard 50 μl qPCR reaction volumes reduces the impact of variations in pipetting and may be preferable for some operators.

**Table 1:** Species quantification results from a survey of selected xenografts.

Xenograft Name	Tumor Type	in vivo Passage	Initial DNA (ng)	Average % Human ± SD	Average % Mouse	Calculated Human DNA	Calculated Mouse DNA	Sum DNA (ng)	% of Initial DNA
HL-60(TB)	Leukemia	P01	27.6	100.0 ± 0.00	0.0	26.96	0.00	27.0	97.7
MDA-MB-361	Breast	P01	48.8	12.8 ± 1.84	87.2	4.80	31.20	36.0	73.7
NCI-H157	Lung	P01	72.4	98.9 ± 1.50	1.1	97.83	1.29	99.1	136.8
RXF-631	Renal	P01	23.2	43.2 ± 0.06	56.8	9.90	11.34	21.2	91.5
SK-MEL-2	Melanoma	P01	29.2	81.8 ± 1.42	18.2	38.63	7.66	46.3	158.4
UACC-257	Melanoma	P01	53.7	99.9 ± 0.08	0.1	49.49	0.04	49.5	92.3
LOX IMVI	Melanoma	P01-a	50.6	48.4 ± 7.26	51.6	16.15	17.54	33.7	66.6
LOX IMVI	Melanoma	P01-b	52.8	55.1 ± 6.38	45.0	19.30	16.77	36.1	68.3
LOX IMVI	Melanoma	P01-c	55.5	39.6 ± 7.99	60.4	17.13	27.34	44.5	80.2
LOX IMVI	Melanoma	P01-d	52.1	53.1 ± 6.56	46.9	24.81	35.35	60.2	115.4
LOX IMVI	Melanoma	P01-e	53.5	33.9 ± 7.76	66.1	20.15	45.56	65.7	122.9
									
AS283	Lymphoma	P04-a	56.9	83.5 ± 5.89	16.5	37.12	7.39	44.5	78.3
AS283	Lymphoma	P04-b	71.6	83.6 ± 4.90	16.4	86.85	13.31	100.2	139.8
CA46	Lymphoma	P04	43.4	99.9 ± 0.09	0.1	47.76	0.04	47.8	110.2
CP70	Ovarian	P04	61.4	83.5 ± 3.23	16.6	59.56	9.87	69.4	113.0
Daudi	Lymphoma	P04	56.5	100.0 ± 0.02	0.0	49.03	0.01	49.0	86.7
GTL-16	Gastric	P04	42.3	100.0 ± 0.00	0.0	39.92	0.00	39.9	94.4
OVCAR-5	Ovarian	P04	53	88.8 ± 0.31	11.2	63.11	6.98	70.1	132.1
									
AS283	Lymphoma	P10	49.2	99.3 ± 0.82	0.7	48.04	0.22	48.3	98.1
HuH-7	Liver	P10	55.2	90.1 ± 4.26	9.9	52.24	4.36	56.6	102.5
NCI-H1975-Luc-GFP	Lung	P10	56.4	34.9 ± 6.18	65.1	18.67	29.45	48.1	85.4
NCI-H209-Luc-GFP	Lung	P10	61.7	81.0 ± 2.00	19.0	79.70	15.83	95.5	154.8

The xenograft names, tumor types, and number of in vivo passages a sample has progressed through are given for each sample at the time of sample collection. Letters following the passage number signify independent tumor samples derived from tumors grown on different mice of the same cohort. After DNA extraction and purification, samples were diluted between 25 ng/μl and 75 ng/μl; one microliter of this dilution serves as the initial DNA (ng) template in the qPCR reactions. The CT value for each sample was derived from standard curves and converted to a log genomes value, which can be converted to the number of measured haploid genomes present. Percent Human and Percent Mouse values were derived by dividing # human genomes by total genomes present or # mouse genomes divided by total genomes present, respectively. Genome numbers for each species are back-calculated into DNA (ng), the sum of which should be equal to the initial DNA used in the qPCR sample. The far right column lists the percentage of the initial sample calculated from the qPCR assay and gives a measure of quality assurance.

## Conclusions

This qPCR method allows for quick and accurate estimations of mouse contamination in human xenografts. This is important when accessing anti-tumor drug efficacy in human tumor xenografts and in evaluating any other mixed DNA sample where significant proportions of mouse DNA could influence the interpretation of the results.

## Methods

### Cell lines and xenografts

Human cancer cell lines were obtained from ATCC/LGC (Wesel, Germany) or the DCTD Repository (Frederick, MD) and were cultured in RPMI Medium 1640 (Life Technologies, Carlsbad, CA) supplemented with 10% fetal calf serum and 2 mM L-glutamine. All cell lines were maintained in a humidified incubator at 37°C in the presence of 5% CO_2_. Human xenograft samples were implanted subcutaneous in athymic nude mice (Balb/c nu/nu, 4-6 weeks old), which were purchased from Charles River Laboratories (Frederick, MD) and maintained under sterile and controlled conditions of temperature (22-24C), light (12-h light/12-h dark), and humidity (45-65%), with food and water ad libitum. Xenografts used for this study were routinely harvested when they reached 500 mg in size, before a necrotic core develops, and after 1, 4, or 10 serial in vivo passages. Each of the fresh tumors harvested for DNA extraction were divided using surgical scissors into multiple fragments of similar size (~150 mg), each containing regions from both the tumor's core and periphery.

### DNA extraction and purification

Tissues and cell lines were processed using a modified version of the REDExtract-N-Amp Tissue PCR kit protocol (Sigma-Aldrich, St. Louis, MO). Briefly, samples (~150 mg wet weight) were incubated overnight in a mixture of 100 μL extraction buffer, 10 μL tissue preparation solution, and 5 μL of 10 mg/ml proteinase K (Life Technologies) at 50°C in a slowly rotating rotisserie oven. Samples were neutralized the next morning by adding 110 μL neutralization buffer and 5 μL 20 mg/mL Purelink RNase-A (Life Technologies), followed by incubations at 37°C and 96°C for 15 minutes each. To quantify DNA for real-time qPCR assays, genomic DNA was purified with the standard phenol-chloroform method and resuspended in 10 mM Tris-Cl buffer, pH 8.0, or diluted in distilled, sterile water. DNA concentrations were measured on a NanoDrop-1000 (Thermo Fisher Scientific, Inc., Waltham, MA).

### Qualitative, end-point PCR

Unique human and mouse-specific primer pairs, designed using Primer3 software [23]http://frodo.wi.mit.edu/primer3/, rely on species-specific differences (underlined in Figure 1b) in the forward primers to amplify 189 bp fragments of the prostaglandin E receptor 2 (PTGER2) gene. PCR primers were purchased from Applied Biosystems (ABI) by Life Technologies. PCR was performed using neutralized but unpurified tissue/cell lysate on an ABI-2720 Thermocycler (Life Technologies). PCR conditions: 95°C-5 min, 30 cycles of (94°C-45 sec, 60°C-30 sec, 72°C-90 sec), 72°C-10 min. DNA bands were resolved on a 2% agarose gel + ethidium bromide (0.5 μg/ml).

### Real-Time Quantitative PCR (qPCR)

The qPCR primers and probes were designed using Primer3 software [23] (http://frodo.wi.mit.edu/primer3/) and purchased from ABI (Life Technologies). Target sequences represent regions located in the human and mouse prostaglandin E receptor 2 (PTGER2) genes (see Figure 1A). Real-time qPCR was carried out on an ABI-7500 Real Time PCR System (Applied Biosystems) using custom-labelled species-specific probes (ABI) according to the manufacturer's protocol with 50 ng of total genomic DNA (unless otherwise specified) in 20 μL reaction volumes. The qPCR conditions were as follows: 50°C-2 min, 95°C-10 min, 40 cycles of (95°C-15 sec, 60°C-1 min). The human+mouse forward primer and the common reverse primer listed in Figure 1B were added to each qPCR reaction tube to obtain the same final concentrations (200 nM). Both probes (Figure 1C) were also added to each reaction tube for a final concentration of 200 nM. Samples were usually run in triplicate on the same reaction plate. Samples were assayed on at least three different 96-well reaction plates, often by two different operators, before statistical analysis was performed. All ΔRn thresholds were calculated by default from the 7500 ABI software, v 2.0.5.

### Data analysis and statistics

Each qPCR reaction plate requires the presence of the standard curve samples, which contain serial dilutions of mouse-only, human-only, or human+mouse mixed samples of known DNA concentration. Standard curves were developed in Microsoft Excel (Microsoft, Redmond, WA) by graphing the mean Threshold Cycle (CT) on the y-axis versus the log initial genomes on the x-axis. A linear trend line was generated, with the equation of the line used to calculate genome number from Mean CT values. "Percent mouse" was estimated by dividing the number of mouse genomes by the sum of both human plus mouse genomes, multiplied by 100. "Percent human" was calculated similarly. The measured genome number was used to back-calculate the measured mass of each species DNA by applying the following equation to each [24]

$$\text{M }=\;{\text{N}}_{\text{g}}\times \text{1}.0\text{96}{\text{e}}^{-\text{21}}\text{g}∕\text{bp}$$

where M = mass of the haploid genome (in grams), N_g _= number of base pairs (bp) in haploid genome, and g = grams. The mouse genome is estimated to be 2.651 billion bp (as of NCBI genome Build 36.1), while the human genome is estimated to be 3.038 billion bp (as of NCBI genome Build 36.3). Thus, one haploid mouse genome is approximately 2.9 pg, whereas one human haploid genome is approximately 3.33 pg.

## Authors' contributions

SYA designed the primers and probes, optimized and carried out PCR assays, and drafted the manuscript. DJK participated in the DNA extraction and carried out PCR assays. SDB and JPC implanted and harvested the xenograft tissues in vivo. KMD performed all the in vitro work. MGH conceived of the study and helped to draft the manuscript. All authors read and approved the final manuscript.
